# Association between the *PARP1* Val762Ala Polymorphism and Cancer Risk: Evidence from 43 Studies

**DOI:** 10.1371/journal.pone.0087057

**Published:** 2014-01-28

**Authors:** Rui-Xi Hua, He-Ping Li, Yan-Bing Liang, Jin-Hong Zhu, Bing Zhang, Sheng Ye, Qiang-Sheng Dai, Shi-Qiu Xiong, Yong Gu, Xiang-Zhou Sun

**Affiliations:** 1 Department of Oncology, The First Affiliated Hospital of Sun Yat-sen University, Guangzhou, China; 2 General Department of Internal Medicine, The First Affiliated Hospital of Sun Yat-sen University, Guangzhou, China; 3 Department of Molecular Epidemiology and Laboratory Medicine, The Affiliated Tumor Hospital of Harbin Medical University, Harbin, China; 4 Department of Nuclear Medicine, the First Affiliated Hospital of Sun Yat-sen University, Guangzhou, China; 5 Department of Pathology, University of Cambridge, Cambridge, United Kingdom; 6 Department of Thoracic Surgery, the First Affiliated Hospital of Sun Yat-sen University, Guangzhou, China; 7 Department of Urinary Surgery, the First Affiliated Hospital of Sun Yat-sen University, Guangzhou, China; Sanjay Gandhi Medical Institute, India

## Abstract

**Background:**

Poly (ADP-ribose) polymerase-1 (PARP-1) plays critical roles in the detection and repair of damaged DNA, as well as cell proliferation and death. Numerous studies have examined the associations between *PARP1* Val762Ala (rs1136410 T>C) polymorphism and cancer susceptibility; nevertheless, the findings from different research groups remain controversial.

**Methods:**

We searched literatures from MEDLINE, EMBASE and CBM pertaining to such associations, and then calculated pooled odds ratio (OR) and 95% confidence interval (CI) by using random-effects model. The false-positive report probability (FPRP) analysis was used to confirm the validity of significant findings. Moreover, potential effects of rs1136410 variants on *PARP1* mRNA expression were analyzed for three ethnicities by combining data from HapMap (genotype) and SNPexp (mRNA expression).

**Results:**

The final meta-analysis incorporated 43 studies, consisting of 17,351 cases and 22,401 controls. Overall, our results did not suggest significant associations between Ala variant (Ala/Ala or Ala/Val genotype) and cancer risk. However, further stratification analysis showed significantly increased risk for gastric cancer (Ala/Ala vs. Val/Val: OR = 1.56, 95% CI = 1.01–2.42, Ala/Val vs. Val/Val: OR = 1.34, 95% CI = 1.14–1.58, dominant model: OR = 1.41, 95% CI = 1.21–1.65 and Ala vs. Val: OR = 1.29, 95% CI = 1.07–1.55). On the contrary, decreased risk for brain tumor (Ala/Val vs. Val/Val: OR = 0.77, 95% CI = 0.68–0.87, dominant model: OR = 0.77, 95% CI = 0.68–0.87 and Ala vs. Val: OR = 0.82, 95% CI = 0.74–0.91). Additionally, we found that the Ala carriers had a significantly increased risk in all models for Asians. Our mRNA expression data provided further biological evidence to consolidate this finding.

**Conclusions:**

Despite some limitations, this meta-analysis found evidence for an association between the *PAPR1* Val762Ala and cancer susceptibility within gastric cancer, brain tumor and Asian subgroups.

## Introduction

The global burden of cancer keeps rising, mainly due to aging and growth of the populations throughout the world, cancer-causing behaviors such as smoking and drinking, as well as environment pollution. As a result, cancer has been recognized as one of the leading cause of death worldwide now. According to the estimation of GLOBOCAN, approximately 12.7 million new cases and 7.6 million deaths of cancer had occurred in 2008. It's noteworthy that about 56% of new cases and 63% of deaths took place in the economically developing countries [1]. The cancer survival tends to be poorer in the developing countries than in the developed countries, most likely due to late stage at diagnosis combined with limited access to timely and standard treatment. The burden of cancer can be largely lessened through the application of early detection and treatment, tobacco control, vaccine injection, healthier dietary intake and so on [2]. Cancer can be initiated by DNA damage caused by exposure to a variety of environmental agents, including UV, ionizing radiation, genotoxic chemicals and products derived from oxidative respiration as well as products of lipid peroxidation that can cause DNA structure alterations. However, the incidence of cancer is relatively low, since humans have developed a set of DNA repair systems to safeguard the integrity of genome by repairing harmful DNA damage. Therefore, DNA repair capacity plays important roles in maintaining the stability and integrity of human genome [3].

In humans, there exist at least four DNA repair pathways, composed of over 130 genes. One of the four pathways, base excision repair (BER) pathway, is responsible for the repair of damaged DNA resulting from exposure to various endogenous and exogenous carcinogens. This pathway primarily removes incorrect and damaged bases, and can specifically remove methylated, oxidized, or reduced single base pair alterations [4]. It has been verified that numerous proteins are involved in the BER pathway, one of which is poly (ADP-ribose) polymerase family member 1 (PARP1) that is also known as adenosine diphosphate ribosyl transferase (ADPRT) [5].

The *PARP1* gene lies in chromosome 1q41-q42, encoding a 113 KDa zinc-finger DNA binding protein—poly (ADP ribosyl) transferase, which can modify various nuclear proteins by poly (ADP-ribosyl)ation [6]. Genetic variations in DNA repair genes can modulate DNA repair capacity to result in accumulation of DNA damage, consequently leading to programmed cell death or unregulated cell growth and cancer [7]. There are at least 1287 reported single nucleotide polymorphisms (SNPs) within the *PARP1* gene, including 202 coding-region single nucleotide polymorphisms (cSNPs). Among all cSNPs of the *PARP1* gene, one of the most investigated SNP is Val762Ala polymorphism (rs1136410 T>C) with minor allele frequency (MAF) >0.05. The very SNP is located in the sixth helix of the catalytic domain, and can cause Val to Ala amino acid substitution at codon 762 of exon 17. Previous studies demonstrated that the *PARP1* Val762Ala polymorphism was related to functional alteration of PARP1, and the Ala allele could significantly reduce poly (ADP-ribosyl)ation activities of PARP1 in an allele dosage-dependent manner [7]. To date, many studies have explored the association between *PARP1* Val762Ala polymorphism and caner risk [7]–[45]; however, the results were inconsistent. The discrepancies among studies may be ascribed to the facts that sample size in each publication was probably relatively small, and that conclusions might have been drawn from different ethnic groups. Hence, we performed the present updated meta-analysis with addition of newly published studies on such association to further elucidate the role of the *PARP1* Val762Ala polymorphism in cancer susceptibility.

## Materials and Methods

### Literature search strategy

We first searched literatures from MEDLINE and EMBASE using the following terms “*PARP* or *PARP1* or *PARP-1* or *poly (ADP-ribose) polymerase 1* or *ADPRT* or *ADPRT1* or *ADPRT 1*”; “polymorphism or variant or variation”; “cancer or carcinoma or tumor or neoplasia” (the last search update on July 28, 2013). We also searched publications written in Chinese from Chinese Biomedical (CBM) database (http://cbmwww.imicams.ae.cn/cbmbin) (1978–) using the combinations terms of “*PARP1*”, “polymorphism” and “cancer” in Chinese to expand the coverage of our current study. Additional relevant studies in the references, such as review articles, original studies were also manually searched. We only included studies with full texts available. Only the latest study or studies with the largest sample size were included in our final meta-analysis to avoid duplication or overlapping data.

### Selection and exclusion criteria

Studies included had to meet the following criteria: evaluate the association between *PARP1* Val762Ala polymorphism and cancer risk; case-control study design; sufficient information for estimating odds ratios (ORs) and their 95% confidence intervals (CIs); independent from other studies; written in English or Chinese; additionally, genotype frequencies data in the controls for Val762Ala departure from Hardy-Weinberg equilibrium (HWE) without further evidence from other SNPs were excluded in the our final analysis.

### Data extraction

Two authors (Rui-Xi Hua and He-Ping Li) independently extracted the following information from each study: the first authors' surname, year of publication, country of origin, ethnicity, cancer type, control source, genotyping methods, total numbers of cases and controls, numbers of cases and controls with the Val/Val, Val/Ala, and Ala/Ala genotypes for *PARP1* Val762Ala polymorphism, minor allele frequency (MAF), *P* value for HWE, and disagreement was resolved by discussions by these two author until consensus was reached. For studies including subjects of different racial descents, data were extracted separately for each ethnic group (categorized as Asian or Caucasian or African).

### Genotype and gene expression correlation analysis

The genotype and mRNA expression levels data for *PARP1* Val762Ala (rs1136410 T>C) were available from HapMap (http://hapmap.ncbi.nlm.nih.gov/) and SNPexp (http://app3.titan.uio.no/biotools/tool.php?app=snpexp), respectively, as described previously [46]–[50]. The genotype data for *PARP1* Val762Ala were retrieved from the HapMap phase II release 23 data set, which consist a total of 3.96 million SNP genotypes derived from 270 individuals of three ethnicities. The mRNA expression data were obtained by performing genome-wide expression arrays for EBV-transformed lymphoblastoid cell lines that were derived from the same 270 individuals.

### Statistical methods

The associations between *PARP1* Val762Ala polymorphism and cancer risk were evaluated by crude ORs and their corresponding 95% CIs for each study. Pooled ORs and 95% CIs for *PARP1* Val762Ala were calculated under homozygous model (Ala/Ala vs. Val/Val), heterozygous model (Val/Ala vs. Val/Val), recessive model [Ala/Ala vs. (Val/Ala & Val/Val)], dominant model [(Val/Ala & Ala/Ala) vs. Val/Val], and allele comparing (Ala vs. Val).

Goodness-of-fit chi-square test was performed to test deviation from HWE and a *P* value less than 0.05 was considered significant. Chi square-based Q-test was used to assess the homogeneity of studies. The fixed-effects model (the Mantel–Haenszel method) [51] was chosen when studies were homogeneous (with *P*>0.10 for the Q test); otherwise, random-effects model (the DerSimonian and Laird method) was adopted [52]. Heterogeneity was also tested by the *I^2^* statistic, with 0% indicating no observed heterogeneity, and larger values indicating increases in heterogeneity [53]. Subgroup analyses were conducted according to cancer type, ethnicity and source of control. Standard error of log (OR) for each study was plotted against its log (OR) to evaluate the potential publication bias. Funnel plot asymmetry was estimated by Egger's linear regression test [54]. Sensitivity analyses were performed by excluding each investigation individually and recalculating the pooled estimates and their corresponding 95% CIs to determine the effect of each study on the summary estimate. The differences in mRNA expression levels among genotypes were tested by one way ANOVA, and the mRNA expression level trends among genotypes were evaluated using General linear model.

To avoid false positive findings, the false-positive report probability (FPRP) values and statistical powers were also calculated for all significant findings observed in the current meta-analysis [55]–[57]. FPRP values with prior probabilities of 0.25, 0.1, 0.01, 0.001 and 0.0001 were obtained, with FPRP value <0.2 considered noteworthy. All statistics were conducted by using STATA version 11.0 (Stata Corporation, College Station, TX) and SAS version 9.1 (SAS Institute, Cary, NC). All *P* values were two-sided, and *P*<0.05 was considered significant.

## Results

### Study characteristics

**Figure 1:**
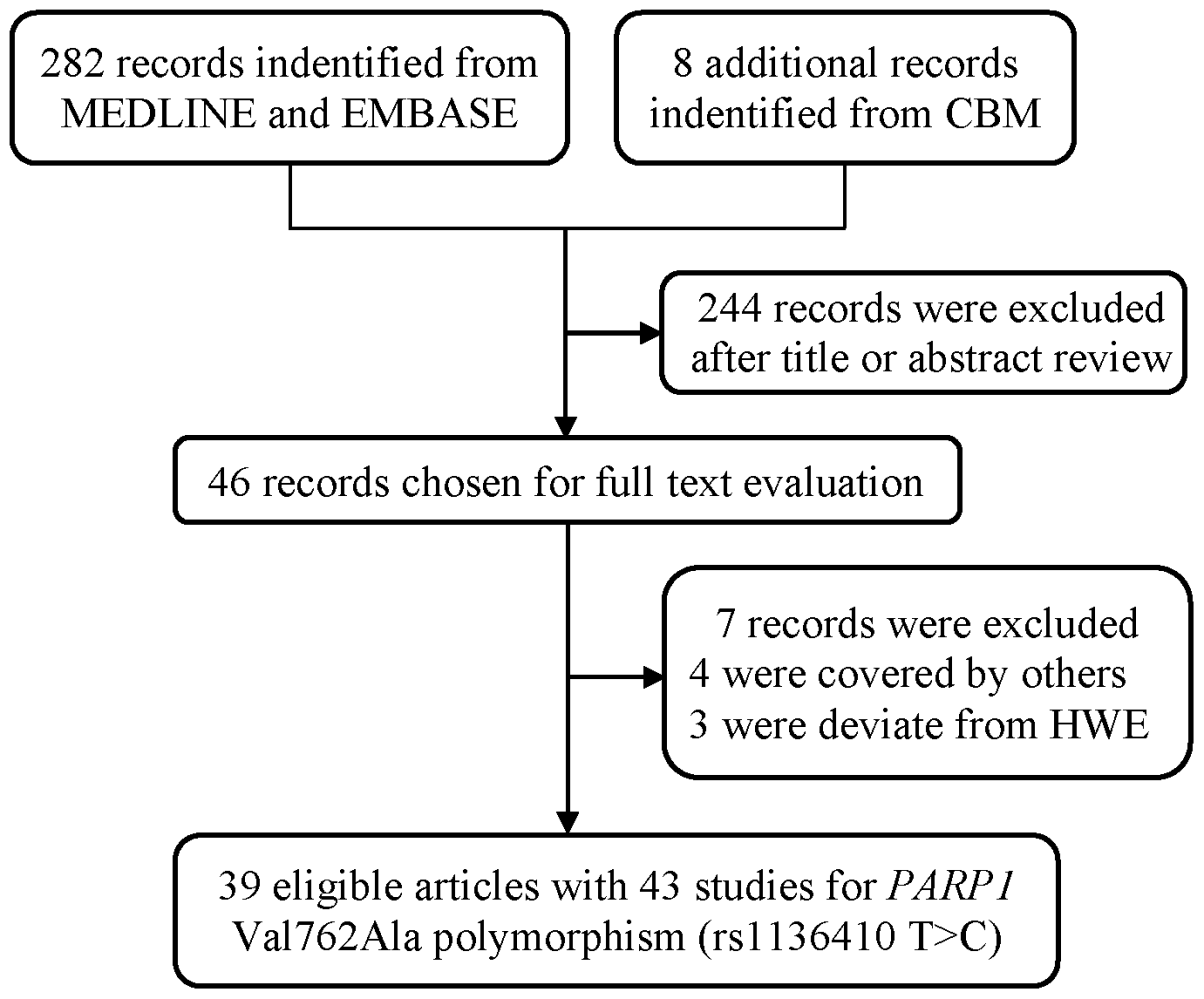
Flow diagram of included studies for the association between *PARP1* Val762Ala polymorphism.

As shown in **Figure 1**, a total of 282 publications were indentified from MEDLINE and EMBASE, and eight additional studies from CBM database. After abstracts and texts assessment, only 46 publications met the crude inclusion criteria and were subjected to further evaluation. Of them, four studies [58]–[60] were excluded for covered by other studies. The genotype distribution of *PARP1* Val762Ala polymorphism in the controls was in compliance with HWE, except for eight studies [13], [19], [34], [37], [41], [61]–[63]. In order to enlarge the sample size and minimize the selection bias, five of these studies [13], [19], [34], [37], [41] were incorporated in our final analysis, because the genotype distributions of other genes (e.g., *XRCC1* or *APE*) in the controls of those studies were consistent with HWE. Rest of studies [61]–[64], exclusively investigating Val762Ala polymorphism, were excluded from pooled analysis, due to the absence of further evidence to confirm validity of their sampling. Finally, only 39 publications were included for the meta-analysis (**Table 1**).

**Table 1:** Characteristics of the 43 studies included in the meta-analysis for an association between *PARP1* Val762Ala polymorphism and risk of cancers.

Surname	Year	Country	Ethnicity	Cancer type	Control source	Genotyping methods	Cases	Controls	MAF	HWE
Lockett	2004	USA	Caucasian	Prostate	HB	MassARRAY	438	427	0.14	0.532
Lockett	2004	USA	African	Prostate	HB	MassARRAY	50	97	0.05	0.632
Hao	2004	China	Asian	Esophageal	HB	PCR-RFLP	414	479	0.41	0.880
Zhang	2005	China	Asian	Lung	HB	PCR-RFLP	1000	1000	0.39	0.057
Zhai	2006	China	Asian	Breast	HB	PCR-RFLP	302	639	0.43	0.164
Zhang	2006	USA	Caucasian	Breast	PB	TaqMan	1716	1371	0.17	0.071
Wu	2006	USA	Caucasian	Bladder	HB	TaqMan	606	595	0.16	0.618
Miao	2006	China	Asian	Gastric	HB	PCR-RFLP	500	1000	0.36	0.026
Landi	2006	Europe	Caucasian	Lung	HB	APEX	292	307	0.18	0.325
Shen	2006	USA	Caucasian	NHL	PB	TaqMan	455	535	0.17	0.246
Li	2006	USA	Caucasian	Melanoma	HB	PCR-RFLP	602	603	0.17	0.827
Cao	2007	France	Caucasian	Breast	HB	Sequence	83	100	0.14	0.104
Figueroa	2007	Spain	Caucasian	Bladder	HB	TaqMan	1138	1131	0.12	0.130
Berndt	2007	USA	Caucasian	Colorectal	PB	TaqMan	691	702	0.17	0.012
Stern	2007	Singapore	Asian	Colorectal	PB	TaqMan	307	1173	0.43	0.457
Li	2007	USA	Caucasian	SCCHN	HB	PCR-RFLP	830	854	0.16	0.074
Smith	2008	USA	Caucasian	Breast	HB	MassARRAY	314	397	0.17	0.819
Smith	2008	USA	African	Breast	HB	MassARRAY	52	72	0.02	0.857
Chiang	2008	China	Asian	Thyroid	HB	TaqMan	283	469	0.41	0.616
Zhang	2008	China	Asian	Gastric	HB	PCR-RFLP	138	110	0.16	0.114
Liu	2009	USA	Caucasian	Glioma	PB	MassARRAY	372	365	0.19	0.587
McKean	2009	USA	Caucasian	Glioblastoma	HB	MassARRAY	987	1935	0.18	0.501
Rajaraman	2010	USA	Caucasian	Neuroma	HB	TaqMan	65	463	0.18	0.970
Rajaraman	2010	USA	Caucasian	Meningioma	HB	TaqMan	121	463	0.18	0.970
Rajaraman	2010	USA	Caucasian	Glioma	HB	TaqMan	340	464	0.18	0.804
Wang	2010	China	Asian	Bladder	HB	PCR-RFLP	234	253	0.44	0.771
Kang	2010	China	Asian	Gastric	PB	SNaPshot	150	152	0.26	0.089
Gao	2010	USA	Caucasian	Prostate	HB	Sequence	453	119	0.19	0.133
Jin	2010	Korea	Asian	NHL	PB	PCR-HRM	573	721	0.45	0.845
Ye	2010	China	Asian	Colorectal	HB	MassARRAY	122	157	/	/
Brevik	2010	USA	Caucasian	Colorectal	FB	TaqMan	308	361	0.19	0.880
Yosunkaya	2010	Turkey	Caucasian	Glioma	HB	PCR-RFLP	119	180	0.34	0.046
Kim	2011	Korea	Asian	Gastric	HB	APEX	151	320	0.43	0.635
Zhang	2011	USA	Caucasian	Melanoma	PB	Illumina	213	205	0.04	0.561
Nakao	2012	Japanese	Asian	Pancreatic	HB	TaqMan	185	1465	0.40	0.012
Santonocito	2012	Italy	Caucasian	Melanoma	PB	Real-time PCR	167	99	0.11	0.238
Santos	2012	Portugal	Caucasian	Thyroid	HB	TaqMan	108	216	0.11	0.066
Wen	2012	China	Asian	Gastric	HB	MassARRAY	307	307	0.44	0.024
Yuan	2012	China	Asian	HNC	HB	TaqMan	395	883	0.42	0.895
Zhang	2012	China	Asian	Cervical	HB	SNPstream	80	176	0.46	0.508
Li	2013	China	Asian	Colorectal	HB	PCR-RFLP	451	626	0.39	0.078
Roszak	2013	Poland	Caucasian	Cervical	PB	HRM	446	491	0.15	0.066
Tang	2013	China	Asian	Breast	HB	MassARRAY	793	845	0.43	0.694

HB, Hospital based; PB, Population based; FB, Family based; NHL, non-Hodgkin lymphoma; SCCHN, Squamous cell carcinoma of the head and neck; HNC, Head and neck cancer; PCR-RFLP, Polymerase chain reaction-restriction fragment length polymorphism; APEX, Arrayed primer extension; HRM, High resolution melting; MAF, Minor allele frequency; HWE, Hardy-Weinberg equilibrium.

Studies including multiple ethnicities [7], [22] or multiple types of cancers [27] were considered as multiple studies. The study carried out by Ye et al.[32] only showed estimates in dominant model without presenting genotype count separately. Overall, in this updated meta-analysis investigating the association between *PARP1* Val762Ala polymorphism and cancer risk, 43 studies with a total number of 17351 cases and 22401 controls were included. Of these 43 studies, sample sizes ranged from 50 to 1736 for cases while varying from 72 to 1935 for controls. The final meta-analysis was composed of six studies focused on breast cancer and brain cancer, five studies on gastric cancer, four studies on colorectal cancer, three studies on prostate cancer, bladder cancer and melanoma, the others with no more than two studies. In term of ethnicity, 18 studies were performed among Asians, 23 studies among Caucasians and two studies among Africans. Of these studies, 10 were population-based, 32 were hospital-based and only one was family-based.

### Meta-analysis results

It was found that there was no significant association between *PARP1* Val762Ala polymorphism and overall cancer risk (homozygous model: OR = 1.10, 95% CI = 0.96–1.25; heterozygous model: OR = 1.04, 95% CI = 0.96–1.12, recessive model: OR = 1.07, 95% CI = 0.95–1.20, dominant model: OR = 1.05, 95% CI = 0.97–1.14, and allele comparing: OR = 1.04, 95% CI = 0.98–1.11) (**Table 2**). In the stratification analyses by cancer types, the polymorphism was found to be statistically significantly associated with increased risk of gastric cancer (homozygous model: OR = 1.56, 95% CI = 1.01–2.42; heterozygous model: OR = 1.34, 95% CI = 1.14–1.58, dominant model: OR = 1.41, 95% CI = 1.21–1.65, and allele comparing: OR = 1.29, 95% CI = 1.07–1.55), but decrease risk for brain tumor (heterozygous model: OR = 0.77, 95% CI = 0.68–0.87, dominant model: OR = 0.77, 95% CI = 0.68–0.87, and allele comparing: OR = 0.82, 95% CI = 0.74–0.91). Stratification analyses by ethnicity elucidated that the Ala carriers among Asians have a significantly increased risk of cancer in all genetics models (homozygous model: OR = 1.23, 95% CI = 1.05–1.44; heterozygous model: OR = 1.13, 95% CI = 1.05–1.22, recessive model: OR = 1.14, 95% CI = 1.00–1.30, dominant model: OR = 1.16, 95% CI = 1.07–1.26, and allele comparing: OR = 1.12, 95% CI = 1.04–1.20). However, stratification analyses by source of controls provided no evidence for significant association of Val762Ala with cancer risk.

**Table 2:** Meta-analysis of the association between *PARP1* Val762Ala polymorphism and cancer risk.

Variables	No. of	Homozygous			Heterozygous			Recessive			Dominant			Allele		
	studies	Ala/Ala vs. Val/Val			Val/Ala vs. Val/Val			Ala/Ala vs. (Val/Ala & Val/Val)			(Val/Ala & Ala/Ala) vs. Val/Val			Ala vs. Val		
		OR (95% CI)	P ^het^	I^2^ (%)	OR (95% CI)	P ^het^	I^2^ (%)	OR (95% CI)	P ^het^	I^2^ (%)	OR (95% CI)	P ^het^	I^2^ (%)	OR (95% CI)	P ^het^	I^2^ (%)
All a	43	1.10 (0.96–1.25)	<0.001	50.4	1.04 (0.96–1.12)	<0.000	56.2	1.07 (0.95–1.20)	0.002	43.9	1.05 (0.97–1.14)	<0.001	63.0	1.04 (0.98–1.11)	<0.001	68.4
Cancer type																
Prostate	3	1.24 (0.25–6.17)	0.012	84.1	1.05 (0.82–1.35)	0.936	0.0	1.23 (0.25–5.97)	0.013	83.8	1.08 (0.85–1.38)	0.586	0.0	1.07 (0.80–1.44)	0.222	33.6
Breast	6	0.95 (0.77–1.15)	0.879	0.0	0.96 (0.81–1.13)	0.157	37.4	0.94 (0.79–1.13)	0.928	0.0	0.95 (0.82–1.11)	0.176	34.7	0.97 (0.87–1.07)	0.277	20.8
Bladder	3	0.99 (0.70–1.41)	0.850	0.0	1.10 (0.84–1.44)	0.057	65.0	0.96 (0.70–1.34)	0.818	0.0	1.09 (0.86–1.39)	0.083	59.9	1.07 (0.90–1.26)	0.159	45.6
Gastric	5	1.56 (1.01–2.42)	0.017	66.8	1.34 (1.14–1.58)	0.726	0.0	1.36 (0.88–2.10)	0.005	72.7	1.41 (1.21–1.65)	0.554	0.0	1.29 (1.07–1.55)	0.051	57.6
Melanoma	3	2.21 (0.43–11.43)	0.160	45.4	1.69 (0.69–4.15)	<0.001	90.7	1.80 (0.49–6.70)	0.243	29.4	1.79 (0.70–4.57)	<0.001	91.7	1.78 (0.74–4.24)	<0.001	91.8
Colorectal	4	1.15 (0.77–1.74)	0.037	64.5	1.07 (0.94–1.24)	0.749	0.0	1.11 (0.78–1.59)	0.060	59.5	1.08 (0.95–1.23)	0.420	0.0	1.08 (0.93–1.26)	0.067	58.1
Brain	6	0.83 (0.55–1.25)	0.254	24.0	0.77 (0.68–0.87)	0.691	0.0	0.93 (0.55–1.56)	0.070	51.0	0.77 (0.68–0.87)	0.907	0.0	0.82 (0.74–0.91)	0.531	0.0
Others	12	1.08 (0.88–1.33)	0.020	52.7	1.06 (0.95–1.18)	0.108	35.3	1.05 (0.89–1.23)	0.115	35.4	1.06 (0.94–1.21)	0.009	56.3	1.04 (0.94–1.15)	0.001	64.5
Ethnicity																
Caucasian	23	0.91 (0.75–1.11)	0.149	24.2	0.96 (0.86–1.07)	<0.001	66.4	0.94 (0.77–1.15)	0.117	27.2	0.96 (0.86–1.08)	<0.001	68.9	0.98 (0.88–1.08)	<0.001	69.6
Asian	18	1.23 (1.05–1.44)	0.001	58.4	1.13 (1.05–1.22)	0.627	0.1	1.14 (1.00–1.30)	0.006	52.7	1.16 (1.07–1.26)	0.157	25.4	1.12 (1.04–1.20)	0.002	56.7
African	2	/	/	/	1.64 (0.62–4.37)	0.279	14.7	/	/	/	1.64 (0.62–4.37)	0.279	14.7	1.60 (0.63–4.10)	0.287	11.8
Source of control																
HB	32	1.13 (0.97–1.32)	<0.001	63.3	1.01 (0.93–1.10)	0.002	47.4	1.10 (0.96–1.26)	0.001	51.0	1.02 (0.94–1.12)	<0.001	58.3	1.03 (0.95–1.11)	<0.001	66.1
PB	10	0.96 (0.78–1.19)	0.023	51.8	1.15 (0.95–1.38)	<0.001	74.0	0.95 (0.81–1.11)	0.464	0.0	1.15 (0.95–1.39)	<0.001	76.9	1.11 (0.95–1.30)	<0.001	77.9
FB	1	1.22 (0.54–2.78)	/	/	1.11 (0.80–1.84)	/	/	1.18 (0.52–2.66)	/	/	1.12 (0.81–1.54)	/	/	1.11 (0.84–1.45)	/	/

Het, heterogeneity; HB, Hospital based; PB, Population based; FB, family based.

a One study (Ye, et al. 2010) was included only in the calculation of the dominant model.

To validate the results, the FPRP values at different prior probability levels were calculated for significant findings and shown in **Table 3**. For a prior probability of 0.01, FPRP value was less than 20%, statistical power was 0.980 and FPRP value was 0.046 for heterozygous model; statistical power was 0.831 and FPRP value was 0.002 for dominant model for gastric cancer, and statistical power was 0.987 and FPRP value was 0.003 for heterozygous model; statistical power was 0.991 and FPRP value was 0.002 for dominant model and statistical power was 1.000, FPRP value was 0.014 for allele comparing for brain tumor. Positive associations with the Ala/Ala genotype were observed in the subgroups for Asians at heterozygous (FPRP = 0.090) and dominant models (FPRP = 0.043). Greater FPRP values were observed for other significant findings.

**Table 3:** False-positive report probability values for associations between cancer risk and the frequency of genotypes of *PARP1* gene.

Variables	OR (95% CI)	*P* a	Statistical Power^b^	Prior Probability				
				0.25	0.1	0.01	0.001	0.0001
Homozygous (Ala/Ala vs. Val/Val)								
Gastric cancer	1.56 (1.01–2.42)	0.046	0.982	0.123	0.297	0.823	0.979	0.998
Asian	1.23 (1.05–1.44)	0.011	1.000	0.032	0.090	0.521	0.917	0.991
Heterozygous (Val/Ala vs. Val/Val)								
Gastric cancer	1.34 (1.14–1.58)	0.0005	0.980	0.001	0.004	0.046	0.329	0.831
Brain tumor	0.77 (0.68–0.87)	<0.0001	0.987	0.000	0.000	0.003	0.029	0.227
Asian	1.13 (1.05–1.22)	0.001	1.000	0.003	0.009	0.090	0.500	0.909
Recessive [Ala/Ala vs. (Val/Ala & Val/Val)]								
Asian	1.14 (1.00–1.30)	0.048	1.000	0.126	0.302	0.826	0.980	0.998
Dominant [(Val/Ala & Ala/Ala) vs. Val/Val]								
Gastric cancer	1.41 (1.21–1.65)	<0.0001	0.831	0.000	0.000	0.002	0.017	0.144
Brain tumor	0.77 (0.68–0.87)	<0.0001	0.991	0.000	0.000	0.002	0.021	0.175
Asian	1.16 (1.07–1.26)	0.0005	1.000	0.001	0.004	0.043	0.313	0.820
Allele (Ala vs. Val)								
Gastric cancer	1.29 (1.07–1.55)	0.007	1.000	0.021	0.059	0.409	0.875	0.986
Brain tumor	0.82 (0.74–0.91)	0.0001	1.000	0.000	0.001	0.014	0.122	0.582
Asian	1.12 (1.04–1.20)	0.004	1.000	0.012	0.035	0.284	0.800	0.976

CI, confidence interval; OR, odds ratio.

a Chi-square test was used to calculate the genotype frequency distributions.

b Statistical power was calculated using the number of observations in the subgroup and the OR and *P* values in this table.

### The correlation between the mRNA expression and genotypes

The potential effects of *PARP1* Val762Ala polymorphism on the mRNA expression levels of *PARP1*gene were explored among three ethnic groups. Ala variants were significantly associated with increased mRNA expression levels for *PARP1* gene among Asians (heterozygous: *P* = 0.025 and dominant: *P* = 0.030), but such effects were not found for Caucasians or the Africans **(** **Table 4**).

**Table 4:** *PARP1* mRNA expression by the genotypes of rs1136410T>C (Val762Ala)^a^.

Population	Genotypes	No.	Mean±SD	*P* b	*P*_trend_ c
CEU^d^	TT	56	7.97±0.28		0.908
	TC	21	7.97±0.32	0.957	
	CC	4	8.03±0.31	0.663	
	Dominant	25	7.98±0.31	0.922	
YRI^d^	TT	83	8.07±0.30		0.476
	TC	1	7.86	0.476	
	CC	0	/	/	
	Dominant	1	7.86	0.476	
Asian^d^	TT	23	7.84±0.17		0.097
	TC	42	7.94±0.16	0.025	
	CC	20	7.94±0.23	0.125	
	Dominant	62	7.94±0.18	0.030	
All^d^	TT	162	8.00±0.29		0.308
	TC	64	7.95±0.22	0.113	
	CC	24	7.96±0.24	0.440	
	Dominant	88	7.95±0.22	0.100	

a *PARP1* genotyping data and mRNA expression levels by genotypes were obtained from the HapMap phase II release 23 data from EBV-transformed lymphoblastoid cell lines from 270 individuals.

b Two-side Student's *t* test within the stratum.

c *P* values for the trend test of *PARP1* mRNA expression among 3 genotypes for each SNP from a general linear model.

d There were missing values because genotyping data not available.

### Heterogeneity and sensitivity analyses

Substantial among-study heterogeneities were observed, while calculating risk estimate for the association between *PARP1* Val762Ala polymorphism and overall cancer risk (homozygous model: *P*<0.001, *I* ^2^ = 50.4%; heterozygous model: *P*<0.001, *I* ^2^ = 56.2%; recessive model: *P* = 0.002, *I* ^2^ = 43.9%; dominant model: *P*<0.001, *I* ^2^ = 63.0% and allele comparing: *P*<0.001, *I* ^2^ = 68.4%). Therefore, random-effects model was chosen to generated wider CIs for all genetics models. Moreover, the leave-one-out sensitivity analyses indicated that there was no any study that could alter the pooled ORs obviously (data not shown).

### Publication bias

The shape of the funnel plots seems asymmetry, and the Egger's test for *PARP1* Val762Ala suggested that there was no significant publication bias in the current meta-analysis (homozygous model: *P* = 0.463, heterozygous model: *P* = 0.367, recessive model: *P* = 0.603, dominant model: *P* = 0.319, and allele comparing: *P* = 0.660).

## Discussion

In this updated meta-analysis of 43 studies with 17351 cases and 22401 controls, pooled analysis did not yield significant association between *PARP1* Val762Ala polymorphism and overall cancer risk. However, further stratified analyses revealed that this polymorphism was associated with an increased risk for gastric cancer, but decreased risk for brain tumor. There results were further validated by FPRP analysis. Moreover, the pooled odds ratio for the association between Ala variants (Ala/Ala or Ala/Val genotype) and cancer risk was statistically significant among Asians. Interestingly, it was also found that *PARP1* Val762Ala polymorphism significantly influenced mRNA expression levels of *PARP1*gene in the Asians, but not in the Caucasians or the Africans, which might help to explain our findings that the association between the polymorphism and cancer risk was only found in the Asians.

As so far, there were only two meta-analyses have being investigated the role of *PARP1* Val762Ala polymorphism in overall cancer risk [65], [66]. To the best of our knowledge, with inclusion of 15 additional studies that were absent in the two previous meta-analysis, the current meta-analysis is the most comprehensive study that has evaluated the association of *PARP1* Val762Ala polymorphism with overall cancer risk. In accordance with our finding, no significant association was observed between this polymorphism and overall cancer risk in one meta-analysis by Yu et al. [65], which including 21 studies with a total of 12027 cases and 14106 controls. The stratified analyses indicated that the Ala allele was associated with an increase risk of cancer among Asians, but a decrease risk among Caucasians, for glioma risk in particular. Similarly, the other meta-analysis of 28 publications with 13745 patients and 16947 controls suggested this polymorphism was not significantly associated with overall cancer risk, except for the Chinese population [66]. One of advantages of the current meta-analysis was that the FPRP analysis was performed to preclude probability of false positive results. It is important to conduct FPRP analysis to calculate statistical power and the opportunity to be false positive findings, especially when the sample size in the strata is not large enough, for some findings may be false positive ones due to the reduced sample size as well as weak association in some subgroups, which need further validation in larger investigations. FPRP analysis ensured that this association of the polymorphism with increased risk for the Asians, gastric cancer, and decreased risk for brain tumor was indeed existed in the heterozygous and dominant models.

In the current meta-analysis, the *PARP1* Val762Ala polymorphism seemed to exert opposite effects on the risks of gastric and brain cancer. It remains unclear whether the *PARP1* Val762Ala polymorphism affects cancer risk through the same biological mechanism across different types of cancer or ethnic group. Nevertheless, it was noteworthy that the opposing results on gastric and brain cancer risks were derived from different ethnic groups. Studies on brain tumor were exclusively performed from Caucasians. In contrast, all studies on gastric cancer were from Asians. Nonetheless, a few evidence suggested the *PARP1* Val762Ala polymorphism might play differential roles in Asians and Caucasians. First, frequencies of the minor allele of the *PARP1* Val762Ala polymorphism among controls were about 0.423 and 0.166 for Asians and Caucasians, respectively [65]. The discrepancy in the MAF of *PARP1* Val762Ala polymorphism between ethnicity may slightly shed light on the observation that this polymorphism differentially modulates cancer susceptibility between Asians and Caucasians. The protective effect of *PARP1* Val762Ala polymorphism on brain cancer risk in Caucasian may be associated relative higher Val (T) allele frequency in this ethnic group. Second, we found that *PARP1* Val762Ala polymorphism significantly altered mRNA expression levels of *PARP1* gene in Asians, but not in Caucasians or Africans. The *PARP1* 762Ala (C) allele can significantly decrease poly (ADP-ribo-syl)action activity in a dosage-dependent manner. Moreover, alteration in the catalytic domain of Ala allele may impair enzymatic activity [7].

The *PARP1* gene encodes a 113 KDa DNA-binding protein ADPRT/PARP1 enzyme. The PARP1 enzyme plays essential roles in BER pathway through detection of DNA strand breaks and poly (ADP-ribosyl)ation of nuclear acceptor proteins responsible for DNA repair programs and/or apoptosis decision [67]. It also participates in DNA-damage signaling, DNA recombination, genomic stability, and the transcriptional regulation of tumor suppressor genes (e.g., p53) [68], [69]. Therefore, genetic variations in DNA repair genes that can modulate DNA repair capacity may contribute to cancer susceptibility. The Val762Ala polymorphism located within the COOH-terminal catalytic domain is associated with deficient poly (ADP-ribosyl)ation activity, which may impede DNA repair capacity of the BER, and thereby cause genome instability [7].

Previously, some investigations demonstrated that genetic alteration of the *PARP1* Val762Ala can modulate cancer susceptibility, and that the frequency of the Ala/Ala genotype was significantly higher in patients when compared with controls [7], [9], [13], [24], [43] with one exception, in which its frequency was found to be significantly lower in patients [21]. Nevertheless, the association of Ala variants and cancer risk was not validated by others [8], [10]–[12], [14]–[20], [22]–[38], [40]–[42], [44], [45]. In accordance with most of the previous studies, the current meta-analysis did not provide evidence that individuals with Ala genotype had significant increased risk of developing cancer, when compared with the Val/Val genotype. In the subgroup analysis by cancer type, the *PARP1* Ala genotype was significantly associated with gastric cancer and brain tumor which may be ascribed to the cancer specificity and sample size. It was also found the Asians had a relatively higher risk of cancer than the Caucasians which may be due to ethnicity difference.

Several limitations of this updated meta-analysis should be considered, though it was strengthened by including the latest publication as well as studies written in Chinese. First, when all eligible data were pooled together, significantly heterogeneities were observed across studies. The results should be interpreted cautiously. Second, lack of the original data and inclusion of only one SNP may hinder the further assessment of gene-gene and gene-environment interactions. Third, the sample size of most included studies is relatively small (<500 for cases) except for 11 studies [9], [11]–[13], [16], [18], [19], [21], [26], [31], [45]. Forth, our results were derived based on unadjusted estimates. A more precise analysis should have been conducted, if individual data such as age, gender, race, smoking and drinking status, pack-years, and environmental factors were available. Finally, since various genotyping methods were adopted across studies, different quality control issues and genotyping bias may be inevitable.

Overall, this updated meta-analysis with addition of fifteen latest published studies allowed us to provide a more precise relative risk estimate regarding the association between *PARP1* Val762Ala polymorphism and cancer susceptibility. These findings suggested that the *PARP1* Val762Ala polymorphism may play a role in cancer development, at least in Asian group or some specific cancer types. For instance, our results showed increased risk of gastric cancer, but decrease risk of brain tumor for Ala carriers, indicating this polymorphism may exert different effects across different types of cancer.

## Supporting Information

Checklist S1. (DOC)
